# Intervenciones para el control de *Aedes aegypti* en América Latina y el Caribe: revisión sistemática y estudio cualitativo

**DOI:** 10.26633/RPSP.2017.17

**Published:** 2017-03-06

**Authors:** Ariel Bardach, Agustín Ciapponi, Andrea Alcaraz, Herney Andrés García-Perdomo, Ruth Amanda Ruano Gándara, María Belizán, Elena Tapia López, Silvina Ruvinsky

**Affiliations:** 1 Centro Cochrane - Instituto de Efectividad Clínica y Sanitaria (IECS) Consejo Nacional de Investigaciones Científicas y Técnicas (CONICET) Argentina Centro Cochrane - Instituto de Efectividad Clínica y Sanitaria (IECS), Consejo Nacional de Investigaciones Científicas y Técnicas (CONICET), Argentina; 2 Instituto de Efectividad Clínica y Sanitaria (IECS) Instituto de Efectividad Clínica y Sanitaria (IECS) Argentina Instituto de Efectividad Clínica y Sanitaria (IECS), Argentina; 3 Universidad del Valle Universidad del Valle Cali Colombia Universidad del Valle, Cali, Colombia

**Keywords:** Control de vectores, Aedes, análisis cualitativo, prevención y control, revisión sistemática, América Latina

## Abstract

**Antecedentes.:**

Al momento no se ha logrado sintetizar toda la información cualicuantitativa relacionada al control de Aedes aegypti (A. aegypti) en América Latina y el Caribe (ALC).

**Objetivo.:**

Describir la existencia y el grado de ejecución de los programas específicos o actividades de control vectorial en ALC como parte de programas sanitarios, establecer los costos y/o costoefectividad de las estrategias de control vectorial e identificar barreras y facilitadores para la implementación de las estrategias.

**Métodos.:**

El estudio se llevará a cabo en dos fases complementarias. La primera fase será cuantitativa en la forma de una revisión sistemática, cuyos detalles han sido publicados en la base PROSPERO (CRD42016038067). La segunda fase será cualitativa y consistirá en la realización de entrevistas en profundidad semiestructuradas a informantes clave como investigadores, responsables programáticos,referentes nacionales, agentes del sistema sanitario y representantes de organizaciones no gubernamentales.

**Discusión.:**

El abordaje cualicuantitativo permitirá describir las estrategias y el nivel de implementación para el control de vector y su efectividad, sus costos programáticos y costo-efectividad. Permitirá también analizar factores influyentes en la implementación de programas.

*Aedes aegypti* (*A. aegypti*) es el principal vector de zika, dengue, chikungunya y fiebre amarilla. Está presente en ámbitos urbanos y silvestres en casi todos los países de la Región de las Américas, excepto Canadá y Chile continental (1). En 2015 aumentaron los casos de insuficiencia placentaria, retraso en el crecimiento, microcefalia, muerte fetal y síndrome de Guillain-Barré asociados a la infección por el virus del zika durante el embarazo (2).

Los macrodeterminantes responsables de la dispersión de la enfermedad son la falta de programas eficaces de control vectorial, el aumento de la densidad poblacional, las malas condiciones sanitarias, el deterioro de los sistemas de salud pública y factores ambientales (3).

Existen estudios sobre prevención y control del dengue; sin embargo, se requieren estudios de mayor calidad metodológica (4, 5). Por otro lado, hasta el momento no se han realizado investigaciones mixtas que logren sintetizar toda la información cualicuantitativa relacionada al control de *A. aegypti* en América Latina y el Caribe (ALC).

## MÉTODOS

El protocolo de la revisión sistemática y todos sus detalles metodológicos se encuentra disponible en el sitio web del Registro prospectivo internacional de revisiones sistemáticas PROSPERO (6).

### Muestreo, recolección y análisis de datos cualitativos

Mediante un muestreo teórico intencional se seleccionarán actores clave en al menos siete países de ALC con carga de enfermedad y programas en marcha (Argentina, Brasil, Colombia, Costa Rica, Cuba, Panamá y Perú han manifestado voluntad de participar). Se realizarán entrevistas en profundidad semiestructuradas a los ejecutores de programas de estos países hasta alcanzar la saturación del discurso. Se utilizará un cuestionario previamente pilotado, administrado por un investigador entrenado en métodos cualitativos. Mediante la guía de preguntas, se buscará entender las fallas en los sistemas de salud que crean barreras a la implementación, así como identificar los facilitadores y las soluciones potenciales para superarlas. También se incluirán consideraciones de género en cuanto a la distribución de las enfermedades y los grupos participantes de programas.

Todas las entrevistas serán grabadas, previo consentimiento informado, y luego transcritas. Un cientista social analizará los datos mediante análisis temático. Los códigos de datos se desarrollarán en base a los temas de la guía de entrevista y se complementarán con códigos adicionales identificados mediante el uso de un enfoque basado en la teoría fundamentada. El análisis se llevará a cabo en las siguientes etapas: factores contextuales, análisis de la entrevista, análisis del grupo objetivo, entre las comparaciones de grupos objetivo, y la comparación entre países. Para la codificación y como soporte para el análisis, se utilizará el programa ATLAS.ti®.

El estudio fue sometido al Comité de ética de la Universidad del Valle, Cali, Colombia.

**Resultados esperados**: Los resultados esperados al finalizar el estudio son:

- Descripción de las estrategias para el control del vector.
- Niveles de implementación (alto, medio y bajo) de las intervenciones por jurisdicción (país y región dentro de país), entendidos como penetrancia del programa en la comunidad.
- Efecto de las intervenciones de control vectorial mediante la utilización de índices vectoriales como índice de recipientes, índice de Breteau, índice de pupas, productividad de recipientes, estimación de población de adultos, índice de contenedores, positividad de tanques, pupas por índice persona, presencia de individuos inmaduros de *A. aegypti* y tasa de positividad en ovitrampas; además de incidencia de enfermedades transmitidas por *A. aegypti*.
- Costos programáticos y datos de costo-efectividad.
- Barreras y facilitadores para la implementación de programas.

## DISCUSIÓN

La evidencia sobre la efectividad programas de control vectorial en la región es escasa y no concluyente (5). Este estudio profundizará la búsqueda de evidencia en ALC complementándola con un abordaje cualitativo que permitirá describir las estrategias y el nivel de implementación para el control de vector, su efectividad, sus costos programáticos y costo-efectividad e identificar barreras y facilitadores en la implementación de programas. Los resultados de la investigación brindará información relevante para la toma de decisiones en la Región.

## Agradecimientos.

Los autores desean agradecer a Daniel Comandé, bibliotecario del Instituto de Efectividad Clínica y Sanitaria (IECS) por su ayuda en el diseño y conducción de la estrategia de búsqueda y a Daniel Salomón, Director del Instituto Nacional de Medicina Tropical (Argentina), por la revisión crítica de este trabajo.

## Conflicto de intereses.

Ninguno declarado por los autores.

## Declaración.

Las opiniones expresadas en este manuscrito son responsabilidad del autor y no reflejan necesariamente los criterios ni la política de la *RPSP/ PAJPH* y/o de la OPS.
